# Supplementary material for: Cell-type differential targeting of SETDB1 prevents aberrant CTCF binding, chromatin looping, and cis-regulatory interactions
Source: Nat Commun. 2024 Jan 2;15:15. doi: 10.1038/s41467-023-44578-0 (PMC10762014; doi:10.1038/s41467-023-44578-0)
Supplement: Supplementary file 1 — Supplementary Information [file 41467_2023_44578_MOESM1_ESM.pdf]

Cell-type differential targeting of SETDB1 prevents aberrant CTCF binding, chromatin looping, and *cis*-regulatory interactions

Phoebe Lut Fei Tam, Ming Fung Cheung, Lu Yan Chan and Danny Leung

Supplementary Information

Supplementary Figures 1-6

Supplementary Table 1

Uncropped blots of Supplementary Figure 2d

Supplementary References

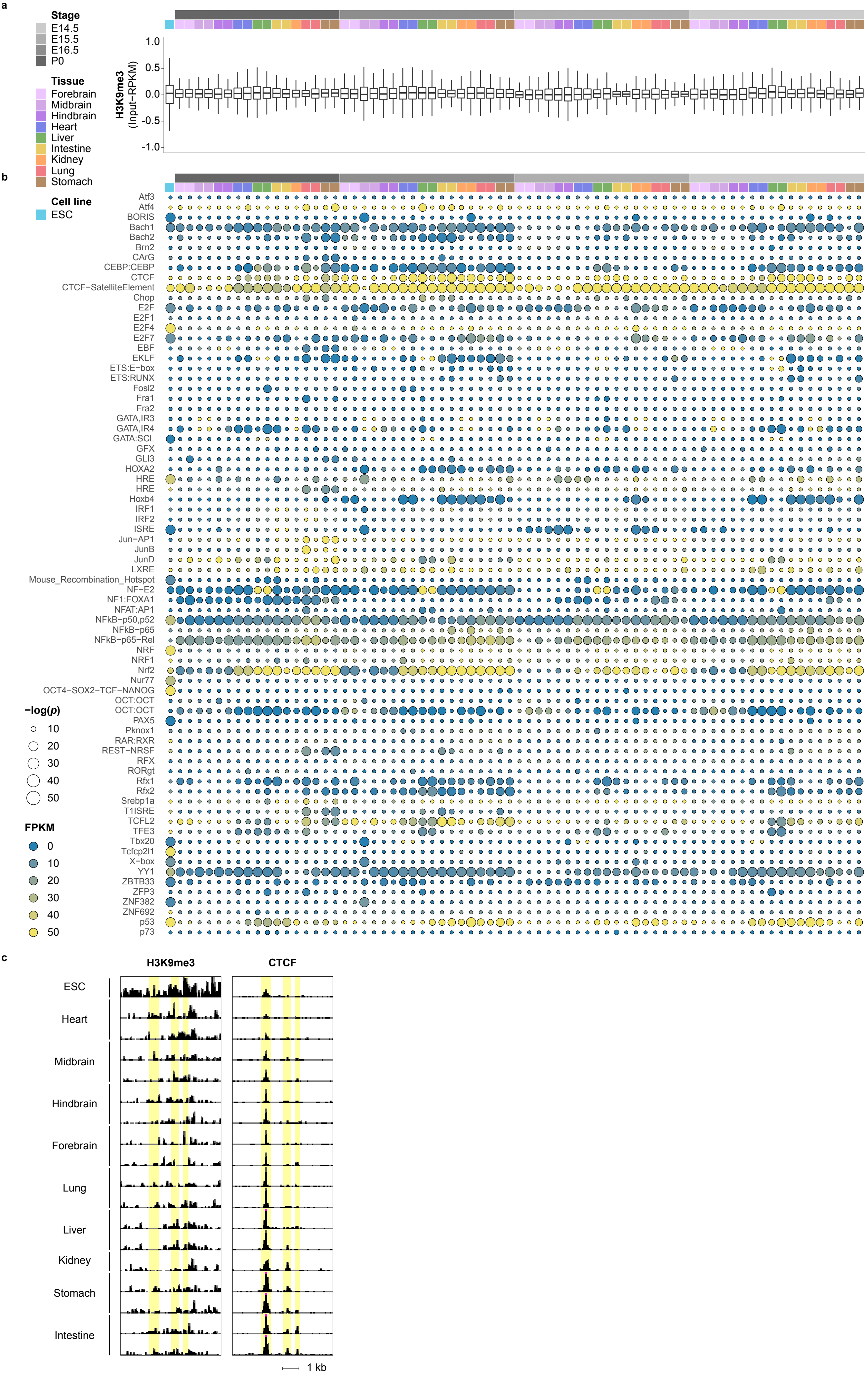
a
Stage
E14.5
E15.5
E16.5
P0
Tissue
Forebrain
Midbrain
Hindbrain
Heart
Liver
Intestine
Kidney
Lung
Stomach
Cell line
ESC
H3K9me3
(Input−RPKM)
1.0
0.5
0.0
-0.5
-1.0
b
Atf3
Atf4
BORIS
Bach1
Bach2
Brn2
CArG
CEBP:CEBP
CTCF
CTCF−SatelliteElement
Chop
E2F
E2F1
E2F4
E2F7
EBF
EKLF
ETS:E−box
ETS:RUNX
Fosl2
Fra1
Fra2
GATA,IR3
GATA,IR4
GATA:SCL
GFX
GLI3
HOXA2
HRE
HRE
Hoxb4
IRF1
IRF2
ISRE
Jun−AP1
JunB
JunD
LXRE
Mouse_Recombination_Hotspot
NF−E2
NF1:FOXA1
NFAT:AP1
NFkB−p50,p52
NFkB−p65
NFkB−p65−Rel
NRF
NRF1
Nrf2
Nur77
OCT4−SOX2−TCF−NANOG
OCT:OCT
OCT:OCT
PAX5
Pknox1
RAR:RXR
REST−NRSF
RFX
RORgt
Rfx1
Rfx2
Srebp1a
T1ISRE
TCFL2
TFE3
Tbx20
Tcfcp2l1
X−box
YY1
ZBTB33
ZFP3
ZNF382
ZNF692
p53
p73
−log(p)
10
20
30
40
50
FPKM
0
10
20
30
40
50
c
H3K9me3
CTCF
ESC
Heart
Midbrain
Hindbrain
Forebrain
Lung
Liver
Kidney
Stomach
Intestine
1 kb

Supplementary Figure 1. Analysis of H3K9me3 enrichment across samples and its relationship with CTCF binding. a) Box plot shows comparable mean H3K9me3 signals (input-RPKM) across the whole genome among each of the analysed samples. **b**) Dot plot represents motif analysis of H3K9me3 peaks conducted by HOMER. Top 20 significant motifs from each sample are included and shown in alphabetical order. Gene expression levels (FPKM) of the corresponding transcription factors in each sample are calculated from publicly available RNA-seq datasets (ENCODE). **c**) A genome browser screenshot demonstrates mutual exclusivity of H3K9me3 (left) and CTCF (right) enrichment in ESCs and tissues at P0. Defined CTCF peaks are highlighted (yellow shading). H3K9me3 ChIP-seq signals are displayed as input subtracted RPKM values with y-axis ranging from 0-10 and 0-5 for ESCs and P0 tissues, respectively. CTCF ChIP-seq signals are shown as input subtracted RPKM values with y-axis ranging from 0-20. Both frames show the same region on chromosome 6 (chr6:37,742,689-37,748,811). Source data are provided as a Source Data file.

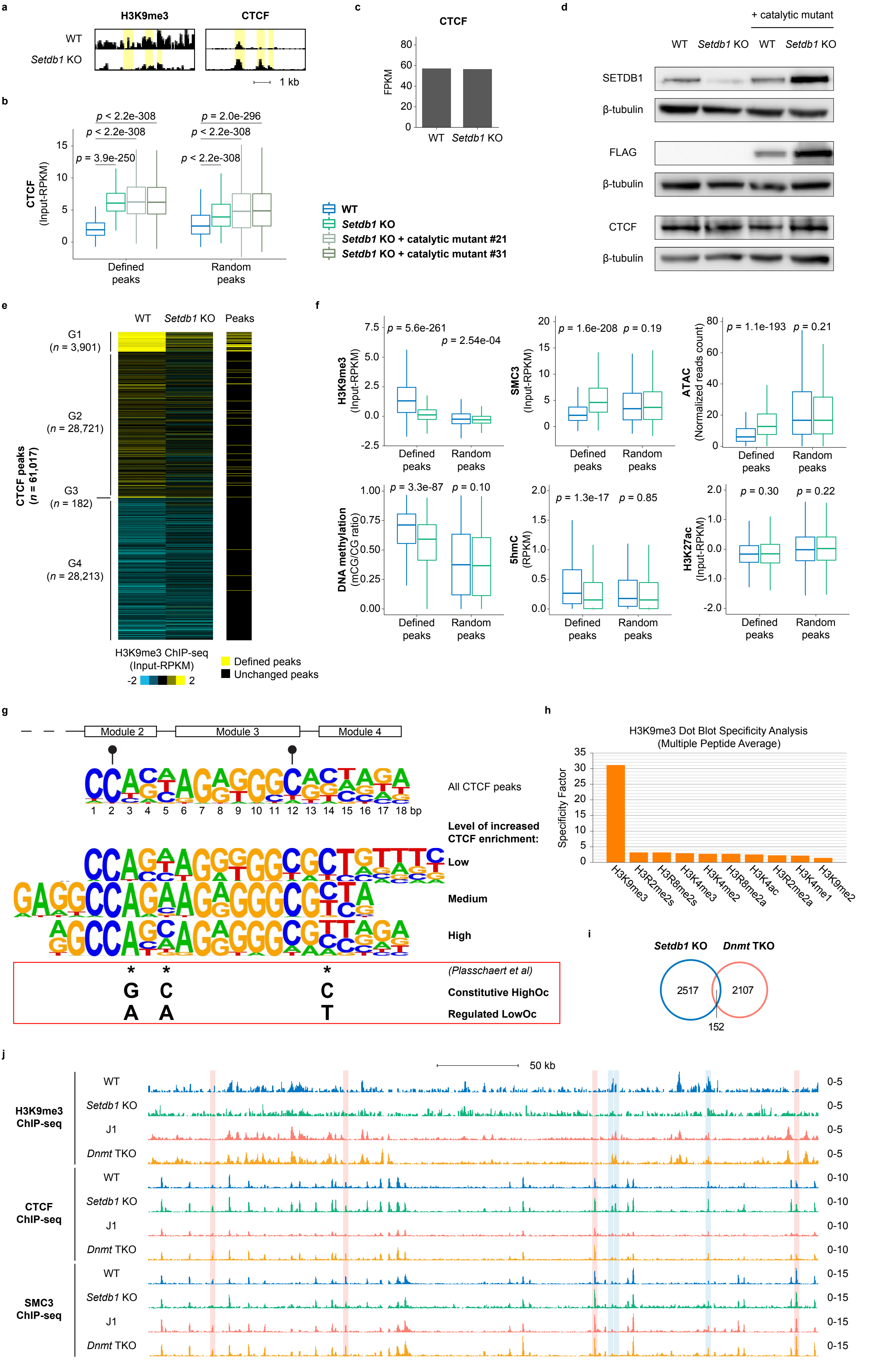
a
H3K9me3
CTCF
WT
Setdb1 KO
1 kb
b
CTCF (Input-RPKM)
p < 2.2e-308
p < 2.2e-308
p = 3.9e-250
p = 2.0e-296
p < 2.2e-308
p < 2.2e-308
Defined peaks
Random peaks
WT
Setdb1 KO
Setdb1 KO + catalytic mutant #21
Setdb1 KO + catalytic mutant #31
c
CTCF
FPKM
WT
Setdb1 KO
d
+ catalytic mutant
WT
Setdb1 KO
WT
Setdb1 KO
SETDB1
β-tubulin
FLAG
β-tubulin
CTCF
β-tubulin
e
WT
Setdb1 KO
Peaks
G1 (n = 3,901)
G2 (n = 28,721)
G3 (n = 182)
G4 (n = 28,213)
CTCF peaks (n = 61,017)
H3K9me3 ChIP-seq (Input-RPKM)
-2
2
Defined peaks
Unchanged peaks
f
H3K9me3 (Input-RPKM)
p = 5.6e-261
p = 2.54e-04
SMC3 (Input-RPKM)
p = 1.6e-208
p = 0.19
ATAC (Normalized reads count)
p = 1.1e-193
p = 0.21
DNA methylation (mCG/CG ratio)
p = 3.3e-87
p = 0.10
5hmC (RPKM)
p = 1.3e-17
p = 0.85
H3K27ac (Input-RPKM)
p = 0.30
p = 0.22
Defined peaks
Random peaks
g
Module 2
Module 3
Module 4
All CTCF peaks
1 2 3 4 5 6 7 8 9 10 11 12 13 14 15 16 17 18 bp
Level of increased CTCF enrichment:
Low
Medium
High
(Plasschaert et al)
Constitutive HighOc
Regulated LowOc
G C C
A A T
h
H3K9me3 Dot Blot Specificity Analysis (Multiple Peptide Average)
Specificity Factor
H3K9me3
H3R2me2s
H3R8me2s
H3K4me3
H3K4me2
H3R8me2a
H3K4ac
H3R2me2a
H3K4me1
H3K9me2
i
Setdb1 KO
Dnmt TKO
2517
2107
152
j
50 kb
H3K9me3 ChIP-seq
CTCF ChIP-seq
SMC3 ChIP-seq
WT
Setdb1 KO
J1
Dnmt TKO
0-5
0-10
0-15

k

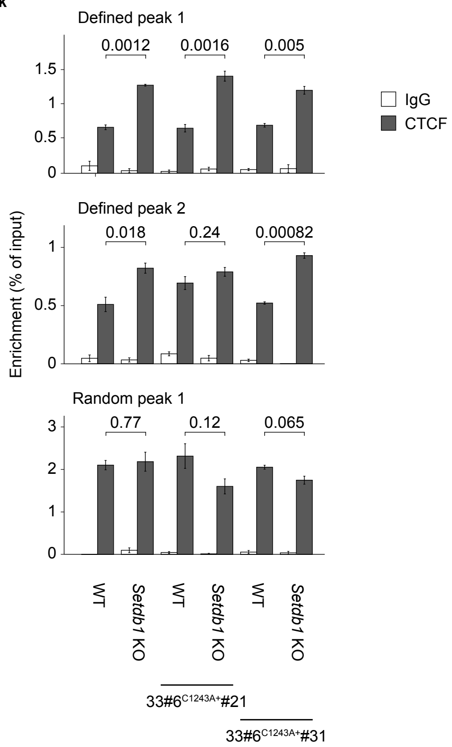
Defined peak 1
0.0012
0.0016
0.005
IgG
CTCF
Defined peak 2
0.018
0.24
0.00082
Enrichment (% of input)
Random peak 1
0.77
0.12
0.065
WT
Setdb1 KO
WT
Setdb1 KO
WT
Setdb1 KO
33#6C1243A+#21
33#6C1243A+#31

Supplementary Figure 2. SETDB1/H3K9m3 regulates CTCF binding independently of DNA methylation.

a) A genome browser screenshot displays an example of increased CTCF binding upon H3K9me3 depletion. H3K9me3 (left) and CTCF (right) ChIP-seq signals are shown as input subtracted RPKM with y-axis are set from 0-10 and 0-20, respectively. The shown region is identical to the chromosome 6 region included in Extended Data Fig. 1c. **b**) Box plots illustrate CTCF ChIP-seq signals (input-RPKM) at increased and number-matched random CTCF peaks (n = 2,669) in WT, *Setdb1* KO, and two lines of SETDB1 catalytic mutant ESCs. p value is calculated by two-tailed Wilcoxon test. The centre and bounds of boxes refer to the median and quartiles of all data points, respectively. The minima and maxima of boxplots indicate Quartile 1 − 1.5 × interquartile range and Quartile 3 + 1.5 × interquartile range, respectively. **c**) CTCF shows similar expression levels by RNA-seq[1] (FPKM) in WT and *Setdb1* KO ESCs. **d**) Western blot results validate the drastic loss of SETDB1 but no detectable changes in CTCF protein abundance in *Setdb1* KO compared to WT ESCs. It also confirms the presence of FLAG-tagged catalytic mutant version of SETDB1 protein. β-tubulin is used as a loading control. **e**) Heatmap generated by k-means clustering (k = 4) shows majority of increased CTCF peaks (mostly in group 1 and 2) exhibit loss of H3K9me3. ChIP-seq signals are displayed as input subtracted RPKM. Increased CTCF peaks are annotated as yellow on the right. **f**) Box plots illustrate H3K9me3, SMC3, and H3K27ac ChIP-seq signals (input-RPKM), 5hmC capture-seq signals (RPKM)[2], DNA methylation levels (mCG/CG ratio)[2], and ATAC-seq signals (normalized reads count) at increased and number-matched random CTCF peaks (n = 2,669) in WT and *Setdb1* KO ESCs. p value is calculated by two-tailed Wilcoxon test. The centre and bounds of boxes refer to the median and quartiles of all data points, respectively. The minima and maxima of boxplots indicate Quartile 1 − 1.5 × interquartile range and Quartile 3 + 1.5 × interquartile range, respectively. **g**) Low occupancy CTCF sites are more susceptible to be regulated by SETDB1. Black circles represent potential DNA methylation at cytosine located at 2nd and 12th bp in a subset of CTCF motifs[3]. Motif logos generated from HOMER of the increased CTCF enrichment sites ranging from lowest to highest. Red box illustrates the 3 base variances of the consensus CTCF motif that enriched in constitutive high occupancy (HighOc), and regulated low occupancy (LowOc), respectively. **h**) Dot blot of H3K9me3 antibody on MODified histone peptide array shows the affinity to distinct histone modifications. The specificity factor is calculated from multiple peptide average. The antibody used has significantly higher affinity to H3K9me3 than any other tested marks. **i**) Venn diagram illustrates the low overlap of increased CTCF peaks between *Setdb1* KO (n = 2,669) and *Dnmt* TKO (n = 2,259) ESCs. **j**) A genome browser screenshots shows examples of *Setdb1* KO-(green shading) and *Dnmt* TKO-specific (orange shading) increased of CTCF binding sites on

chromosome 11 (chr11:114,520,000-114,920,000). A mild global increase of CTCF binding were observed in both *Setdb1* KO and *Dnmt* TKO. H3K9me3, CTCF, and SMC3 ChIP-seq signals are shown as input subtracted RPKM. **k**) SETDB1 catalytic mutant ESCs shows the same trend of increased CTCF occupancy. ESCs were analysed using ChIP with specific for CTCF and non-specific IgG (IgG) as a control. Quantitative PCR (qPCR) was carried out using primers specific for increased (defined) and random CTCF peaks defined in *Setdb1* KO ESCs. Values are presented as relative to input. Error bars represent ± standard deviation between technical triplicates. Significance was measured by Student's t-test. Source data are provided as a Source Data file.

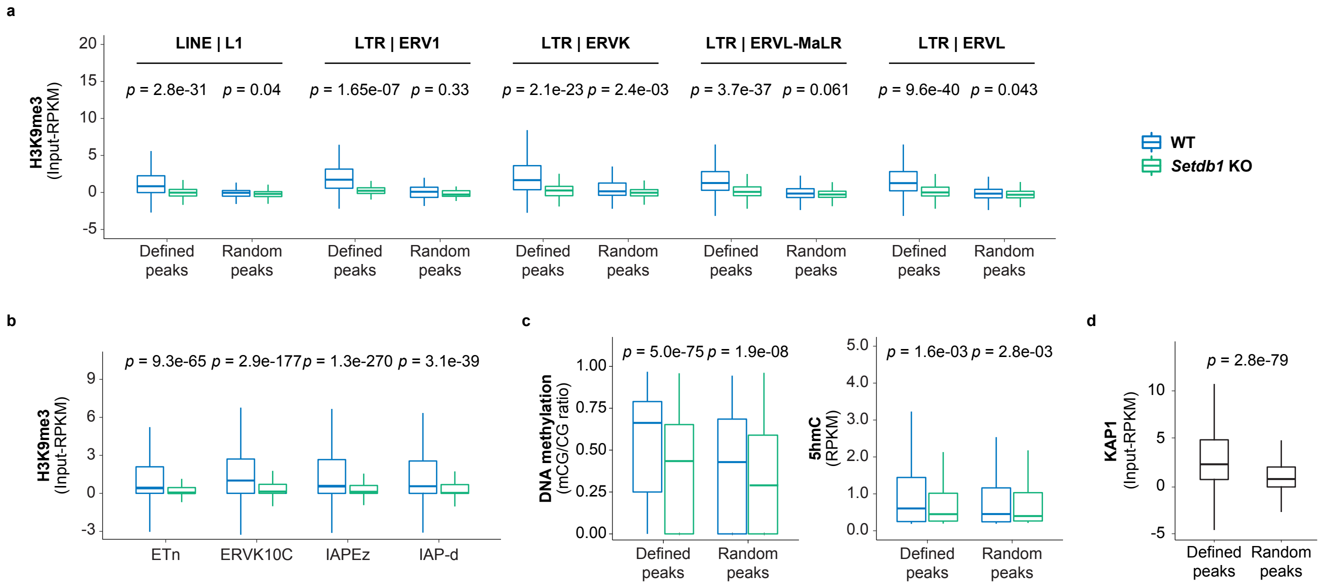
a
H3K9me3 (Input-RPKM)
LINE | L1
LTR | ERV1
LTR | ERVK
LTR | ERVL-MaLR
LTR | ERVL
p = 2.8e-31 p = 0.04
p = 1.65e-07 p = 0.33
p = 2.1e-23 p = 2.4e-03
p = 3.7e-37 p = 0.061
p = 9.6e-40 p = 0.043
Defined peaks
Random peaks
WT
Setdb1 KO
b
H3K9me3 (Input-RPKM)
p = 9.3e-65 p = 2.9e-177 p = 1.3e-270 p = 3.1e-39
ETn
ERVK10C
IAPEz
IAP-d
c
DNA methylation (mCG/CG ratio)
p = 5.0e-75 p = 1.9e-08
5hmC (RPKM)
p = 1.6e-03 p = 2.8e-03
d
KAP1 (Input-RPKM)
p = 2.8e-79

Supplementary Figure 3. H3K9me3 and DNA methylation profiles of retrotransposons.

a) Box plot shows H3K9me3 ChIP-seq signals (input-RPKM) at L1, ERV1, ERVK, ERVL-MaLR, and ERVL elements located at increased and number-matched random CTCF peaks (n = 2,669) in WT and *Setdb1* KO ESCs, respectively. p value is calculated by two-tailed Wilcoxon test. The centre and bounds of boxes refer to the median and quartiles of all data points, respectively. The minima and maxima of boxplots indicate Quartile 1 − 1.5 × interquartile range and Quartile 3 + 1.5 × interquartile range, respectively. **b**) Box plot shows significant H3K9me3 enrichment (input-RPKM) at known Setdb1-targeted ERVs (ETn: n = 1,805; ERVK10C: n = 3,230; IAPEz: n = 7,319; IAP-d: n = 1,323). p value is calculated by two-tailed Wilcoxon test. The centre and bounds of boxes refer to the median and quartiles of all data points, respectively. The minima and maxima of boxplots indicate Quartile 1 − 1.5 × interquartile range and Quartile 3 + 1.5 × interquartile range, respectively. **c**) Box plots demonstrates 5hmC (RPKM) capture-seq signals[2] and DNA methylation levels[2] (mCG/CG ratio) of B2 elements located at increased and number-matched random CTCF peaks (n = 2,669) in WT and *Setdb1* KO ESCs, respectively. p value is calculated by two-tailed Wilcoxon test. The centre and bounds of boxes refer to the median and quartiles of all data points, respectively. The minima and maxima of boxplots indicate Quartile 1 − 1.5 × interquartile range and Quartile 3 + 1.5 × interquartile range, respectively. **d**) Box plots demonstrates KAP1 ChIP-seq signals[4] (input-RPKM) at SINE B2 elements located at increased and number-matched random CTCF peaks (n = 2,669) in J1 ESCs. p value is calculated by two-tailed Wilcoxon test. The centre and bounds of boxes refer to the median and quartiles of all data points, respectively. The minima and maxima of boxplots indicate Quartile 1 − 1.5 × interquartile range and Quartile 3 + 1.5 × interquartile range, respectively. Source data are provided as a Source Data file.

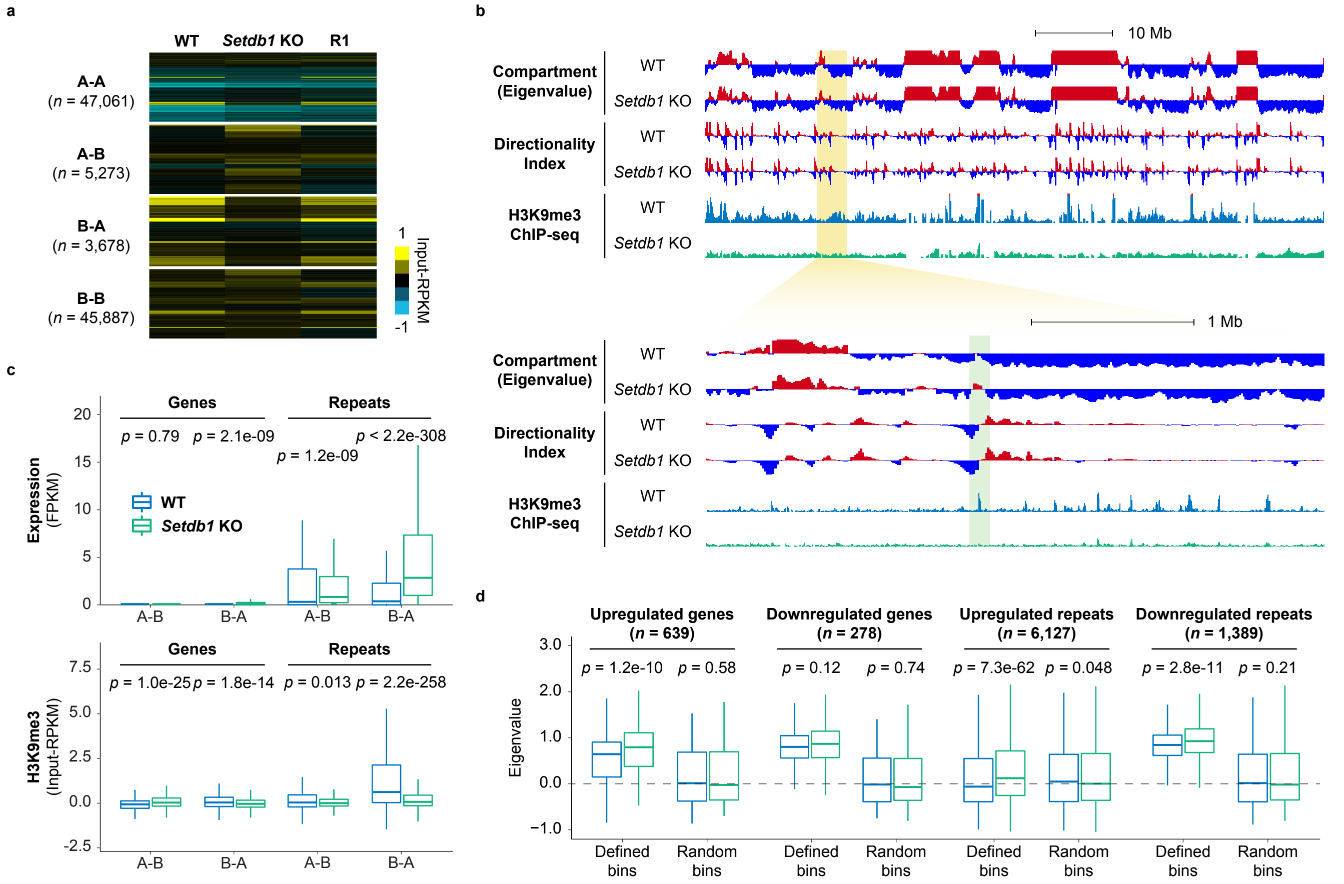

a
WT
Setdb1 KO
R1
A-A
(n = 47,061)
A-B
(n = 5,273)
B-A
(n = 3,678)
B-B
(n = 45,887)
1
-1
Input-RPKM
b
10 Mb
Compartment (Eigenvalue)
Directionality Index
H3K9me3 ChIP-seq
WT
Setdb1 KO
1 Mb
c
Genes
Repeats
p = 0.79
p = 2.1e-09
p = 1.2e-09
p < 2.2e-308
Expression (FPKM)
WT
Setdb1 KO
A-B
B-A
p = 1.0e-25
p = 1.8e-14
p = 0.013
p = 2.2e-258
H3K9me3 (Input-RPKM)
d
Upregulated genes (n = 639)
Downregulated genes (n = 278)
Upregulated repeats (n = 6,127)
Downregulated repeats (n = 1,389)
p = 1.2e-10
p = 0.58
p = 0.12
p = 0.74
p = 7.3e-62
p = 0.048
p = 2.8e-11
p = 0.21
Eigenvalue
Defined bins
Random bins

Supplementary Figure 4. Subtle compartment changes in *Setdb1* KO ESCs.

a) Heatmap shows H3K9me3 signals (input-RPKM) of each bin as demonstrated in Fig. 4a, in WT, *Setdb1* KO, and R1 ESCs. Similar H3K9me3 enrichment was observed in datasets from our WT and from R1[5] cells in B-to-A switching (*n* = 3,678) and unchanged B (*n* = 45,887) compartment bins. A small subset of A-to-B switching bins (*n* = 5,273) show change in *Setdb1* KO cells, while unchanged A compartments (*n* = 47,061) show no substantial H3K9me3 enrichment in any samples. **b**) A genome browser screenshot illustrates consistent compartmentalization (y-axis eigenvalue: -0.5 - 0.5) and TADs (directionality index score: -1000 - 1000) in a large region on chromosome 6 (chr6:724,733-83,490,822) (top). H3K9me3 ChIP-seq signals are shown as input subtracted RPKM (y-axis: 0-2). Zooming into the region (chr6:17,192,714-21,728,783) (bottom), an example is shown of compartment switching from B to A concomitant with H3K9me3 loss (green shading). Notably, the adjacent B compartment bins marked with H3K9me3 display no eigenvalue change in *Setdb1* KO cells. Positive and negative scores represent compartment A and B, respectively. **c**) Box plots illustrate expression and H3K9me3 enrichment of all genes and repeats that are located in compartment-switching bins (genes at A-B: *n* = 1,625; genes at B-A: *n* = 1,893; repeats at A-B: *n* = 523; repeats at A-B: *n* = 5,235) in WT and *Setdb1* KO ESCs. *p* value is calculated by two-tailed Wilcoxon test. The centre and bounds of boxes refer to the median and quartiles of all data points, respectively. The minima and maxima of boxplots indicate Quartile 1 − 1.5 × interquartile range and Quartile 3 + 1.5 × interquartile range, respectively. **d**) Box plot shows subtle changes of eigenvalue of bins that overlapped with dysregulated genes and repeats and number-matched random bins as shown in Fig. 4b. *p* value is calculated by two-tailed Wilcoxon test. The centre and bounds of boxes refer to the median and quartiles of all data points, respectively. The minima and maxima of boxplots indicate Quartile 1 − 1.5 × interquartile range and Quartile 3 + 1.5 × interquartile range, respectively. Source data are provided as a Source Data file.

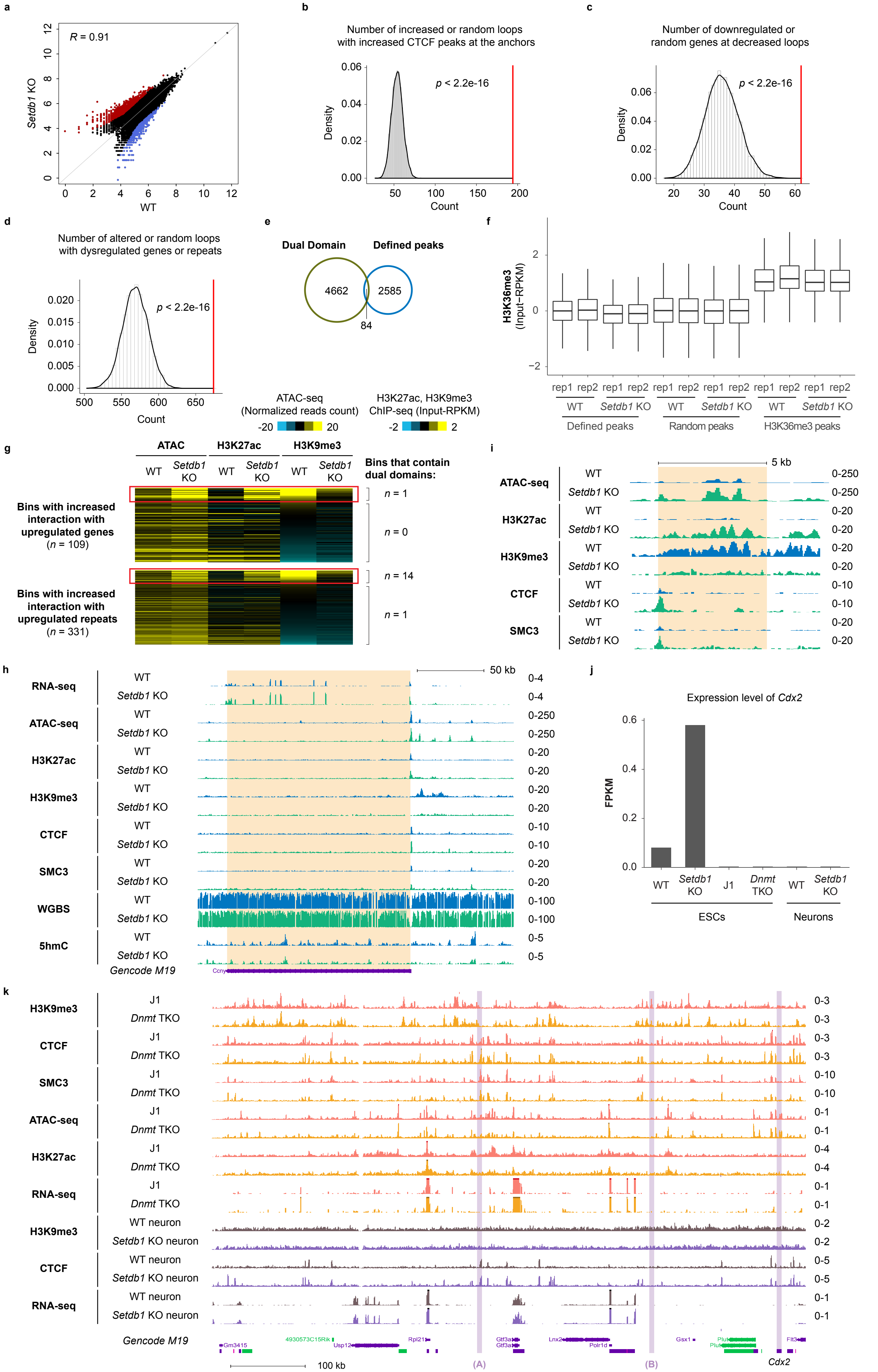

a
R = 0.91
Setdb1 KO
WT
b
Number of increased or random loops
with increased CTCF peaks at the anchors
p < 2.2e-16
Density
Count
c
Number of downregulated or
random genes at decreased loops
p < 2.2e-16
Density
Count
d
Number of altered or random loops
with dysregulated genes or repeats
p < 2.2e-16
Density
Count
e
Dual Domain
Defined peaks
4662
2585
84
f
H3K36me3
(Input−RPKM)
rep1 rep2 rep1 rep2 rep1 rep2 rep1 rep2 rep1 rep2 rep1 rep2
WT
Setdb1 KO
Defined peaks
Random peaks
H3K36me3 peaks
ATAC-seq
(Normalized reads count)
H3K27ac, H3K9me3
ChIP-seq (Input-RPKM)
g
ATAC
H3K27ac
H3K9me3
Bins that contain
dual domains:
Bins with increased
interaction with
upregulated genes
(n = 109)
Bins with increased
interaction with
upregulated repeats
(n = 331)
n = 1
n = 0
n = 14
n = 1
i
5 kb
ATAC-seq
H3K27ac
H3K9me3
CTCF
SMC3
h
50 kb
RNA-seq
ATAC-seq
H3K27ac
H3K9me3
CTCF
SMC3
WGBS
5hmC
Gencode M19
Ccny
j
Expression level of Cdx2
FPKM
WT
Setdb1 KO
J1
Dnmt TKO
ESCs
Neurons
k
H3K9me3
CTCF
SMC3
ATAC-seq
H3K27ac
RNA-seq
J1
Dnmt TKO
WT neuron
Setdb1 KO neuron
Gencode M19
Gm3415
4930573C15Rik
Usp12
Rpl21
Gtf3a
Lnx2
Polr1d
Gsx1
Pluk
Flt3
Cdx2
100 kb
(A)
(B)

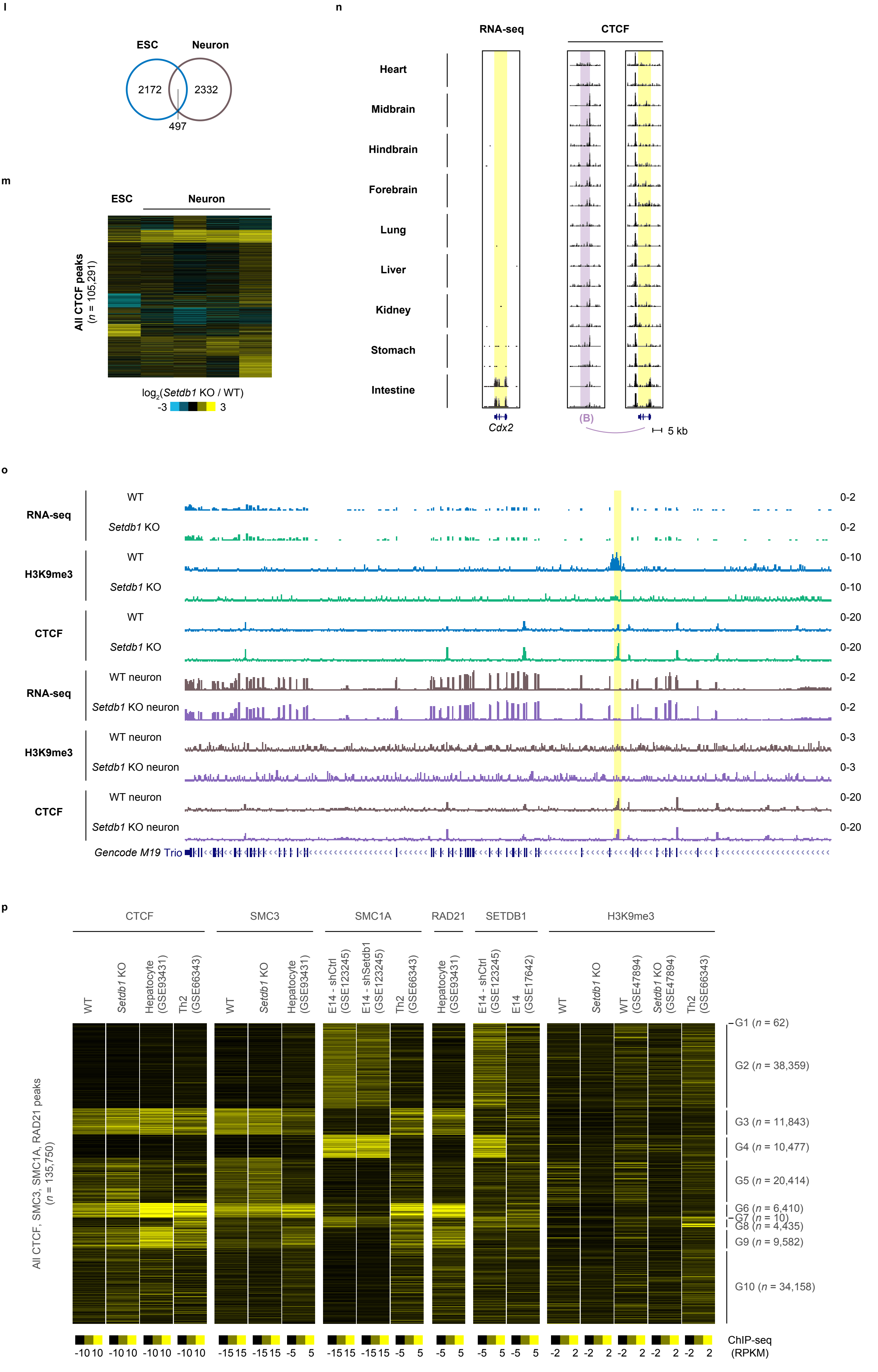

l
ESC
Neuron
2172
2332
497
m
ESC
Neuron
All CTCF peaks
(n = 105,291)
log2(Setdb1 KO / WT)
-3
3
n
RNA-seq
CTCF
Heart
Midbrain
Hindbrain
Forebrain
Lung
Liver
Kidney
Stomach
Intestine
Cdx2
(B)
5 kb
o
RNA-seq
WT
Setdb1 KO
H3K9me3
WT
Setdb1 KO
CTCF
WT
Setdb1 KO
RNA-seq
WT neuron
Setdb1 KO neuron
H3K9me3
WT neuron
Setdb1 KO neuron
CTCF
WT neuron
Setdb1 KO neuron
0-2
0-2
0-10
0-10
0-20
0-20
0-2
0-2
0-3
0-3
0-20
0-20
Gencode M19 Trio
p
CTCF
SMC3
SMC1A
RAD21
SETDB1
H3K9me3
WT
Setdb1 KO
Hepatocyte (GSE93431)
Th2 (GSE66343)
WT
Setdb1 KO
Hepatocyte (GSE93431)
E14 - shCtrl (GSE123245)
E14 - shSetdb1 (GSE123245)
Th2 (GSE66343)
Hepatocyte (GSE93431)
E14 - shCtrl (GSE123245)
E14 (GSE17642)
WT
Setdb1 KO
WT (GSE47894)
Setdb1 KO (GSE47894)
Th2 (GSE66343)
All CTCF, SMC3, SMC1A, RAD21 peaks
(n = 135,750)
G1 (n = 62)
G2 (n = 38,359)
G3 (n = 11,843)
G4 (n = 10,477)
G5 (n = 20,414)
G6 (n = 6,410)
G7 (n = 10)
G8 (n = 4,435)
G9 (n = 9,582)
G10 (n = 34,158)
-10 10 -10 10 -10 10 -10 10
-15 15 -15 15 -5 5
-15 15 -15 15 -5 5
-5 5
-5 5 -5 5
-2 2 -2 2 -2 2 -2 2 -2 2
ChIP-seq
(RPKM)

Supplementary Figure 5. Disrupted chromatin loops and altered transcriptions.

a) Scatter plot shows contact frequencies of all chromatin loops defined by HiCCUPs (n = 16,554). Loops with increased interaction frequencies (q < 0.1 and fold change > 2; n = 794) and decreased interaction frequencies (q < 0.1 and fold change < 0.5; n = 499) in *Setdb1* KO cells are marked with red and blue dots, respectively. Pearson correlation coefficient (R) is included. **b**) Density curve (grey) illustrates the distribution (counts) of increased or number-matched, randomly sampled (10,000 times) chromatin loops (n = 794) that overlap with increased CTCF binding sites at least one anchor. The red line indicates the actual number of loops (n = 193) with increased CTCF peaks. p values are obtained by non-parametric bootstrapping. Related to Fig. 5b. **c**) Density curve (grey) illustrates the distribution (counts) of number-matched randomly selected (10,000 times) genes (n = 286) that are located at decreased chromatin loops. The red line indicates the actual number of downregulated genes that fall into decreased chromatin loops (n = 62). p values are obtained by non-parametric bootstrapping. Related to Fig. 5c. **d**) Density curve (grey) illustrates the distribution (counts) of altered or number-matched, randomly sampled (10,000 times) chromatin loops (n = 1,293) that overlap with dysregulated genes. The red line indicates the actual number of altered loops (n = 674) that contain dysregulated genes. p values are obtained by non-parametric bootstrapping. Related to Fig. 5d. **e**) Venn diagram shows minimal overlap between H3K9me3/H3K36me3 co-enriched dual domains and H3K9me3-regulated CTCF binding sites. **f**) Boxplot illustrates CTCF peaks are H3K36me3-independent. Both H3K9me3-regulated and number-matched random CTCF peaks (n = 2,669) show background levels of H3K36me3 enrichment. H3K36me3 peaks are shown as positive control. The centre and bounds of boxes refer to the median and quartiles of all data points, respectively. The minima and maxima of boxplots indicate Quartile $1 - 1.5 \times$ interquartile range and Quartile $3 + 1.5 \times$ interquartile range, respectively. **g**) Heatmap shows the ATAC-seq signal (normalized read counts) and the H3K27ac and H3K9me3 ChIP-seq signals (input-RPKM) at bins that have increased interactions with non-H3K9me3 marked upregulated genes (n = 109) and repeats (n = 331) in WT and *Setdb1* KO ESCs. Bins are sorted by WT H3K9me3 signal. A subset of bins (red box) show loss of H3K9me3 and gaining of H3K27ac and chromatin accessibility in *Setdb1* KO cells. These may be putative *cis*-regulatory elements for the associated upregulated genes. Number of bins that overlapped with H3K9me3/H3K36me3 co-enriched dual domains is annotated on the right. **h**) A genome browser screenshot shows the upregulated *Ccny* gene (RNA-seq *Setdb1* KO vs WT fold change = 2.35, q = 3.90e-36) with no change in chromatin accessibility, H3K27ac and H3K9me3 enrichment, and DNA methylation level. **i**) A genome browser screenshot shows the putative enhancer region (chr18:9,585,000-9,590,000) (orange shading) that significantly gained interaction (q =

0.00273) with the promoter of *Ccny* shown in panel h. **j**) *Cdx2* shows no derepression in *Dnmt* TKO ESCs and *Setdb1* KO neurons as detected by RNA-seq (FPKM)[6,7]. **k**) A genome browser screenshot illustrates the significantly lost and gained interactions defined in *Setdb1* KO ESCs between *Cdx2* and distal bins (bins A and B, respectively (purple shading)) from Fig. 5a. Bin B shows no gained CTCF binding in *Dnmt* TKO ESCs and *Setdb1* KO neurons and is associated to silenced *Cdx2*. H3K9me3, CTCF, SMC3, and H3K27ac ChIP-seq tracks are displayed as input subtracted RPKM values. ATAC-seq and RNA-seq datasets are shown as RPM values. **l**) Venn diagram illustrates the low overlap of increased CTCF peaks between *Setdb1* KO ESCs (*n* = 2,669) and *Setdb1* KO neurons (*n* = 2,829). **m**) Heatmap generated by *k*-means clustering (*k* = 8) shows differential regulated CTCF enrichment by SETDB1 in mouse ESCs and neurons. Overall increase of CTCF binding in whole genome is observed from both cell-types. **n**) A genome browser screenshot shows P0 mouse intestine to be the only analysed tissue-type with *Cdx2* expression and CTCF binding at both *Cdx2* (yellow shading) and bin B (purple shading). RNA-seq datasets are shown as RPM values with y-axis ranging from 0-5. CTCF ChIP-seq tracks are displayed as input subtracted RPKM values with y-axis ranging from 0-8. **o**) A genome browser screenshot illustrates an example of ESC-specific CTCF regulation by SETDB1 (yellow shading). SETDB1 functions to prevent CTCF binding at this locus in ESCs. However, the sequence is occupied by CTCF in neurons, regardless of *Setdb1* deletion status. RNA-seq datasets are shown as RPM values. H3K9me3 and CTCF ChIP-seq tracks are displayed as input subtracted RPKM values. **p**) Heatmaps show CTCF, SMC3, SMC1A, RAD21, SETDB1, and H3K9me3 ChIP-seq signals (RPKM) in WT and *Setdb1* KO ESCs, Hepatocytes[8], and T helper 2 cells (Th2)[9]. Similar global enrichment patterns of CTCF, SMC3, SMC1A, and RAD21 are observed across cell-types. However, SMC1A and SETDB1 ChIP-seq datasets from Warrier et al are not consistent on the most part[10] (group 2, 4, and 8). Moreover, our H3K9me3 ChIP-seq datasets from WT and *Setdb1* KO ESCs are similar to previously published datasets generated from the same cell line. Source data are provided as a Source Data file.

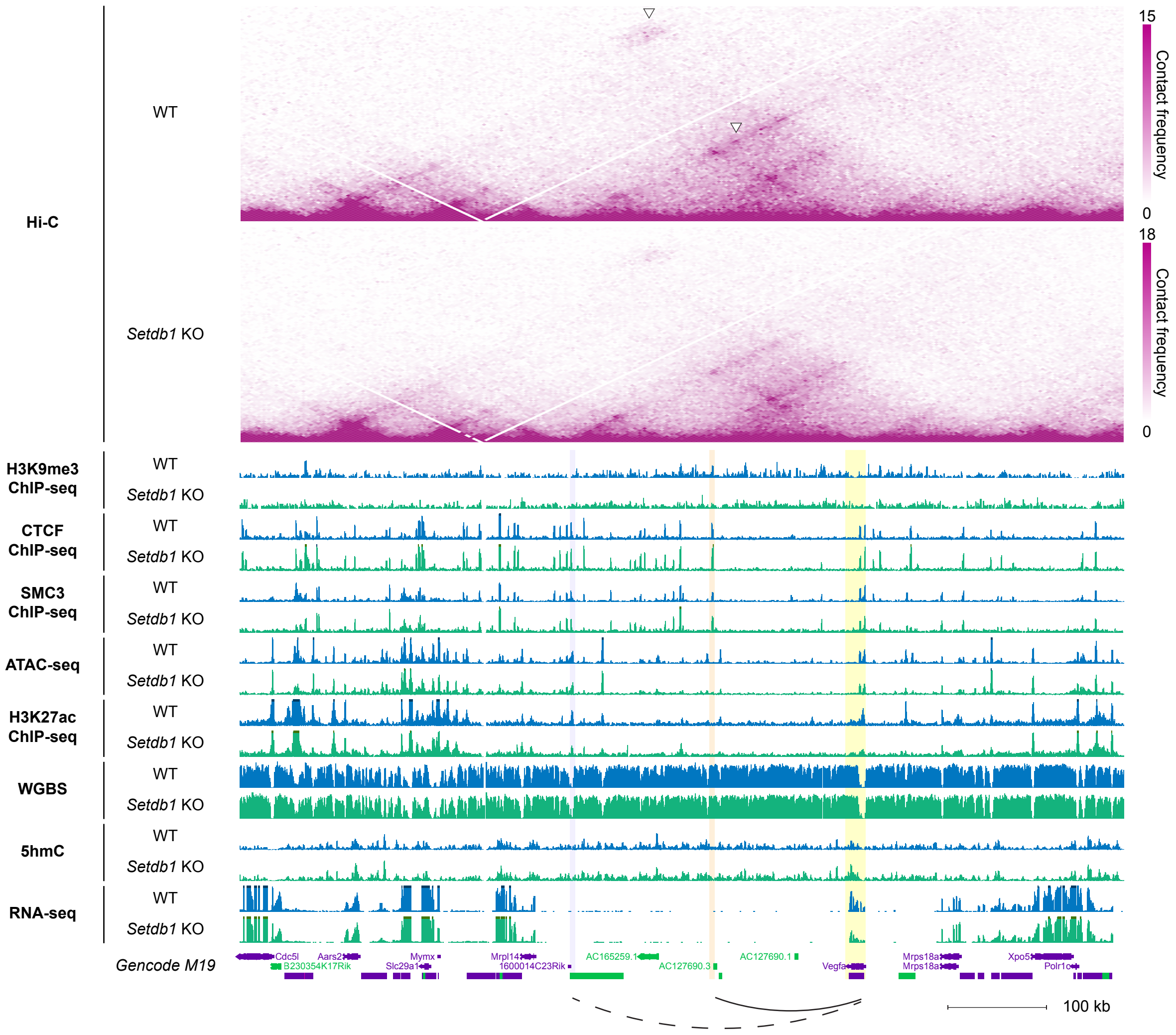
Hi-C
WT
Setdb1 KO
15
0
18
0
Contact frequency
H3K9me3 ChIP-seq
CTCF ChIP-seq
SMC3 ChIP-seq
ATAC-seq
H3K27ac ChIP-seq
WGBS
5hmC
RNA-seq
Gencode M19
Cdc5l
Aars2
B230354K17Rik
Slc29a1
Mymx
Mrpl14
1600014C23Rik
AC165259.1
AC127690.3
AC127690.1
Vegfa
Mrps18a
Xpo5
Polr1c
100 kb

Supplementary Figure 6

A genome browser screenshot illustrates the dynamic interactome of the downregulated *Vegfa* gene (yellow shading) located within decreased loops (white arrowheads) on chromosome 17 (chr17:45,400,000-46,300,000). The solid arc indicates significantly gained interaction between *Vegfa* and a distal bin (orange shading). Conversely, the dashed arc indicates significantly reduced interactions between *Vegfa* and distinct distal bin (purple shading). The originally interacting bin harbours active *cis*-regulatory signatures. Hi-C contact heatmaps display the raw counts in 5 kb windows. H3K9me3, CTCF, SMC3, and H3K27ac ChIP-seq tracks are displayed as input subtracted RPKM values with y-axis ranging from 0-5, 0-5, 0-10 and 0-5, respectively. ATAC-seq tracks are displayed as normalized read counts and y-axis are set from 0-80. WGBS datasets are shown as mCG/CG ratio for CG sites with more than 5 reads coverage and y-axis are set from 0-1. 5hmC datasets are displayed as RPKM values with y-axis are set from 0-10. RNA-seq datasets are shown as RPM values with y-axis ranging from 0-1.5.

Supplementary Table 1. H3K9me3 ChIP-seq peak calling in mouse tissue/cell-types.

A table showing numbers of H3K9me3 peaks called by EPIC v0.2.9 for each sample.

Tissue	Replicate	E14.5	E15.5	E16.5	P0
Forebrain	Rep1	30,719	16,562	40,486	22,891
	Rep2	25,796	21,596	33,723	24,525
Midbrain	Rep1	37,020	21,204	44,309	28,175
	Rep2	24,966	24,541	38,357	21,467
Hindbrain	Rep1	28,545	26,335	51,854	35,037
	Rep2	36,883	30,959	42,150	23,725
Heart	Rep1	55,017	26,363	57,408	37,087
	Rep2	57,853	24,051	55,046	40,026
Liver	Rep1	41,912	33,971	48,662	48,918
	Rep2	53,543	25,755	35,208	52,597
Intestine	Rep1	34,009	18,083	37,697	58,269
	Rep2	30,860	17,345	45,423	19,856
Kidney	Rep1	46,989	31,003	39,797	N/A
	Rep2	26,805	24,973	52,220	15,868
Lung	Rep1	35,533	24,646	43,795	28,400
	Rep2	17,671	16,630	32,973	35,907
Stomach	Rep1	19,920	16,296	34,259	18,009
	Rep2	38,366	18,847	30,203	21,774

Cell Line					
ESC	21,774				

Uncropped blots of Supplementary Figure 2d

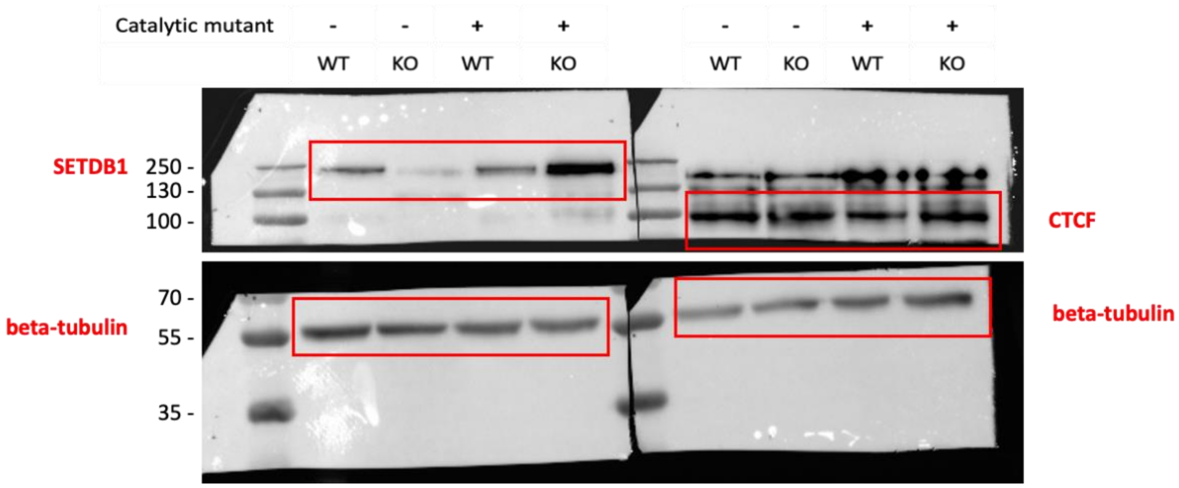

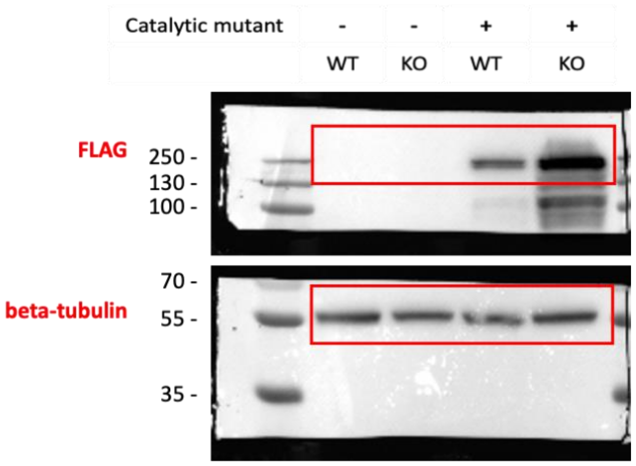

Supplementary References

1. Karimi, M.M. *et al.* DNA methylation and SETDB1/H3K9me3 regulate predominantly distinct sets of genes, retroelements, and chimeric transcripts in mESCs. *Cell Stem Cell* **8**, 676-87 (2011).
2. Leung, D. *et al.* Regulation of DNA methylation turnover at LTR retrotransposons and imprinted loci by the histone methyltransferase Setdb1. *Proc Natl Acad Sci U S A* **111**, 6690-5 (2014).
3. Ong, C.T. & Corces, V.G. CTCF: an architectural protein bridging genome topology and function. *Nat Rev Genet* **15**, 234-46 (2014).
4. Castro-Diaz, N. *et al.* Evolutionally dynamic L1 regulation in embryonic stem cells. *Genes & Development* **28**, 1397-1409 (2014).
5. Kidder, B.L., Hu, G., Cui, K. & Zhao, K. SMYD5 regulates H4K20me3-marked heterochromatin to safeguard ES cell self-renewal and prevent spurious differentiation. *Epigenetics Chromatin* **10**, 8 (2017).
6. Jiang, Q. *et al.* G9a Plays Distinct Roles in Maintaining DNA Methylation, Retrotransposon Silencing, and Chromatin Looping. *Cell Rep* **33**, 108315 (2020).
7. Jiang, Y. *et al.* The methyltransferase SETDB1 regulates a large neuron-specific topological chromatin domain. *Nat Genet* **49**, 1239-1250 (2017).
8. Schwarzer, W. *et al.* Two independent modes of chromatin organization revealed by cohesin removal. *Nature* **551**, 51-+ (2017).
9. Ren, G. *et al.* CTCF-Mediated Enhancer-Promoter Interaction Is a Critical Regulator of Cell-to-Cell Variation of Gene Expression. *Mol Cell* **67**, 1049-1058 e6 (2017).
10. Warrier, T. *et al.* SETDB1 acts as a topological accessory to Cohesin via an H3K9me3-independent, genomic shunt for regulating cell fates. *Nucleic acids research* **50**, 7326-7349 (2022).
