# Supplementary material for: Cell-type differential targeting of SETDB1 prevents aberrant CTCF binding, chromatin looping, and cis-regulatory interactions
Source: Nat Commun. 2024 Jan 2;15:15. doi: 10.1038/s41467-023-44578-0 (PMC10762014; doi:10.1038/s41467-023-44578-0)
Supplement: Supplementary file 3 — Description of Additional Supplementary Files [file 41467_2023_44578_MOESM3_ESM.pdf]

Supplementary Data Legends

File Name: Supplementary Data 1

Description: A table showing the quality control metrics for each Hi-C experimental replicate as generated by Juicer. Also included are the numbers of Hi-C contacts defined in each cell line after merging replicates, which are the definitions used in differential analyses.

File Name: Supplementary Data 2

Description: A table showing non-H3K9me3 marked, dysregulated genes that reside in altered chromatin loops as defined by HiCCUPS. Also included are the altered interactions generated by FitHiC. "v" denotes presence.

File Name: Supplementary Data 3

Description: A table showing publicly available NGS Datasets included in integrative analysis and their sources.
