# Supplementary material for: Cell-type differential targeting of SETDB1 prevents aberrant CTCF binding, chromatin looping, and cis-regulatory interactions
Source: Nat Commun. 2024 Jan 2;15:15. doi: 10.1038/s41467-023-44578-0 (PMC10762014; doi:10.1038/s41467-023-44578-0)
Supplement: Supplementary file 7 — Reporting Summary [file 41467_2023_44578_MOESM7_ESM.pdf]

nature portfolio

Corresponding author(s): Danny Chi Yeu Leung

Last updated by author(s): Nov 6, 2023

Reporting Summary

Nature Portfolio wishes to improve the reproducibility of the work that we publish. This form provides structure for consistency and transparency in reporting. For further information on Nature Portfolio policies, see our Editorial Policies and the Editorial Policy Checklist.

Statistics

For all statistical analyses, confirm that the following items are present in the figure legend, table legend, main text, or Methods section.

n/a	Confirmed	
☐	☒	The exact sample size (*n*) for each experimental group/condition, given as a discrete number and unit of measurement
☐	☒	A statement on whether measurements were taken from distinct samples or whether the same sample was measured repeatedly
☐	☒	The statistical test(s) used AND whether they are one- or two-sided<br>*Only common tests should be described solely by name; describe more complex techniques in the Methods section.*
☒	☐	A description of all covariates tested
☐	☒	A description of any assumptions or corrections, such as tests of normality and adjustment for multiple comparisons
☐	☒	A full description of the statistical parameters including central tendency (e.g. means) or other basic estimates (e.g. regression coefficient) AND variation (e.g. standard deviation) or associated estimates of uncertainty (e.g. confidence intervals)
☐	☒	For null hypothesis testing, the test statistic (e.g. *F*, *t*, *r*) with confidence intervals, effect sizes, degrees of freedom and *P* value noted<br>*Give P values as exact values whenever suitable.*
☒	☐	For Bayesian analysis, information on the choice of priors and Markov chain Monte Carlo settings
☒	☐	For hierarchical and complex designs, identification of the appropriate level for tests and full reporting of outcomes
☐	☒	Estimates of effect sizes (e.g. Cohen's *d*, Pearson's *r*), indicating how they were calculated

Our web collection on statistics for biologists contains articles on many of the points above.

Software and code

Policy information about availability of computer code

Data collection	No software was used for data collection

Data analysis

The software and versions used in this study are as follows:

Bowtie v1.2
Bowtie2 v2.3.3.1, v2.4.2
Picard v2.9.0, v2.23.8
EPIC v0.2.9
MACS2 v2.1.0
HOMER-4.9.1-6
STAR v2.5.3a
Bismark v0.29.0
Trim Galore v0.4.3, v0.6.6
GenomicAlignments v1.18.1
bamtools v2.5.1
DANPOS v2.2.2
deepTools v3.0.1
Juicer v1.5.6
cooltools v0.3.2

Normalization of MNase-seq was done with S3norm. (https://github.com/guanjue/S3norm)

For manuscripts utilizing custom algorithms or software that are central to the research but not yet described in published literature, software must be made available to editors and reviewers. We strongly encourage code deposition in a community repository (e.g. GitHub). See the Nature Portfolio guidelines for submitting code & software for further information.

Data

Policy information about availability of data

All manuscripts must include a data availability statement. This statement should provide the following information, where applicable:

- Accession codes, unique identifiers, or web links for publicly available datasets
- A description of any restrictions on data availability
- For clinical datasets or third party data, please ensure that the statement adheres to our policy

All next-generation sequencing data generated in this study have been deposited to GEO under the accession number GSE184471. All publicly available datasets used and their accession codes are as follows:
H3K9me3 ChIP-seq on mouse forebrain tissue embryo (14.5 days) ENCSR270WWV
H3K9me3 ChIP-seq on mouse forebrain tissue embryo (15.5 days) ENCSR668BBX
H3K9me3 ChIP-seq on mouse forebrain tissue embryo (16.5 days) ENCSR352NVU
H3K9me3 ChIP-seq on mouse forebrain tissue postnatal (0 days) ENCSR093DWU
H3K9me3 ChIP-seq on mouse midbrain tissue embryo (14.5 days) ENCSR948VPV
H3K9me3 ChIP-seq on mouse midbrain tissue embryo (15.5 days) ENCSR864DSI
H3K9me3 ChIP-seq on mouse midbrain tissue embryo (16.5 days) ENCSR571WQI
H3K9me3 ChIP-seq on mouse midbrain tissue postnatal (0 days) ENCSR065LAB
H3K9me3 ChIP-seq on mouse hindbrain tissue embryo (14.5 days) ENCSR689NXE
H3K9me3 ChIP-seq on mouse hindbrain tissue embryo (15.5 days) ENCSR299DRH
H3K9me3 ChIP-seq on mouse hindbrain tissue embryo (16.5 days) ENCSR427ZQB
H3K9me3 ChIP-seq on mouse hindbrain tissue postnatal (0 days) ENCSR365CUP
H3K9me3 ChIP-seq on mouse heart tissue embryo (14.5 days) ENCSR007XTC
H3K9me3 ChIP-seq on mouse heart tissue embryo (15.5 days) ENCSR071SQK
H3K9me3 ChIP-seq on mouse heart tissue embryo (16.5 days) ENCSR122CJZ
H3K9me3 ChIP-seq on mouse heart tissue postnatal (0 days) ENCSR965JWF
H3K9me3 ChIP-seq on mouse liver tissue embryo (14.5 days) ENCSR125VVG
H3K9me3 ChIP-seq on mouse liver tissue embryo (15.5 days) ENCSR855NKG
H3K9me3 ChIP-seq on mouse liver tissue embryo (16.5 days) ENCSR095FDG
H3K9me3 ChIP-seq on mouse liver tissue postnatal (0 days) ENCSR341XCL
H3K9me3 ChIP-seq on mouse intestine tissue embryo (14.5 days) ENCSR698WCJ
H3K9me3 ChIP-seq on mouse intestine tissue embryo (15.5 days) ENCSR311VKI
H3K9me3 ChIP-seq on mouse intestine tissue embryo (16.5 days) ENCSR358XYP
H3K9me3 ChIP-seq on mouse intestine tissue postnatal (0 days) ENCSR672UQX
H3K9me3 ChIP-seq on mouse kidney tissue embryo (14.5 days) ENCSR405TGI
H3K9me3 ChIP-seq on mouse kidney tissue embryo (15.5 days) ENCSR499NUH
H3K9me3 ChIP-seq on mouse kidney tissue embryo (16.5 days) ENCSR821GHK
H3K9me3 ChIP-seq on mouse kidney tissue postnatal (0 days) ENCSR498EVD
H3K9me3 ChIP-seq on mouse lung tissue embryo (14.5 days) ENCSR311LZM
H3K9me3 ChIP-seq on mouse lung tissue embryo (15.5 days) ENCSR773EGV
H3K9me3 ChIP-seq on mouse lung tissue embryo (16.5 days) ENCSR579UVB
H3K9me3 ChIP-seq on mouse lung tissue postnatal (0 days) ENCSR238ZCJ
H3K9me3 ChIP-seq on mouse stomach tissue embryo (14.5 days) ENCSR976GSO
H3K9me3 ChIP-seq on mouse stomach tissue embryo (15.5 days) ENCSR975QEX
H3K9me3 ChIP-seq on mouse stomach tissue embryo (16.5 days) ENCSR630AOU
H3K9me3 ChIP-seq on mouse stomach tissue postnatal (0 days) ENCSR556DLJ
RNA-seq on mouse forebrain tissue embryo (14.5 days) ENCSR185LWM
RNA-seq on mouse forebrain tissue embryo (15.5 days) ENCSR752RGN
RNA-seq on mouse forebrain tissue embryo (16.5 days) ENCSR080EVZ
RNA-seq on mouse forebrain tissue postnatal (0 days) ENCSR362AIZ

RNA-seq on mouse midbrain tissue embryo (14.5 days) ENCSR343YLB
RNA-seq on mouse midbrain tissue embryo (15.5 days) ENCSR557RMA
RNA-seq on mouse midbrain tissue embryo (16.5 days) ENCSR367ZPZ
RNA-seq on mouse midbrain tissue postnatal (0 days) ENCSR719NAJ
RNA-seq on mouse hindbrain tissue embryo (14.5 days) ENCSR559TRB
RNA-seq on mouse hindbrain tissue embryo (15.5 days) ENCSR401BSG
RNA-seq on mouse hindbrain tissue embryo (16.5 days) ENCSR285WZV
RNA-seq on mouse hindbrain tissue postnatal (0 days) ENCSR017JEG
RNA-seq on mouse heart tissue embryo (14.5 days) ENCSR727FHP
RNA-seq on mouse heart tissue embryo (15.5 days) ENCSR597UZW
RNA-seq on mouse heart tissue embryo (16.5 days) ENCSR020DGG
RNA-seq on mouse heart tissue postnatal (0 days) ENCSR526SEX
RNA-seq on mouse liver tissue embryo (14.5 days) ENCSR867YNV
RNA-seq on mouse liver tissue embryo (15.5 days) ENCSR611PTP
RNA-seq on mouse liver tissue embryo (16.5 days) ENCSR826HIQ
RNA-seq on mouse liver tissue postnatal (0 days) ENCSR096STK
RNA-seq on mouse intestine tissue embryo (14.5 days) ENCSR932TRU
RNA-seq on mouse intestine tissue embryo (15.5 days) ENCSR370SFB
RNA-seq on mouse intestine tissue embryo (16.5 days) ENCSR848GST
RNA-seq on mouse intestine tissue postnatal (0 days) ENCSR331XCE
RNA-seq on mouse kidney tissue embryo (14.5 days) ENCSR504GEG
RNA-seq on mouse kidney tissue embryo (15.5 days) ENCSR062VTB
RNA-seq on mouse kidney tissue embryo (16.5 days) ENCSR537GNQ
RNA-seq on mouse kidney tissue postnatal (0 days) ENCSR173PJN
RNA-seq on mouse lung tissue embryo (14.5 days) ENCSR039ADS
RNA-seq on mouse lung tissue embryo (15.5 days) ENCSR457RRW
RNA-seq on mouse lung tissue embryo (16.5 days) ENCSR992WBR
RNA-seq on mouse lung tissue postnatal (0 days) ENCSR982MRY
RNA-seq on mouse stomach tissue embryo (14.5 days) ENCSR290RRR
RNA-seq on mouse stomach tissue embryo (15.5 days) ENCSR906YQZ
RNA-seq on mouse stomach tissue embryo (16.5 days) ENCSR466KZY
RNA-seq on mouse stomach tissue postnatal (0 days) ENCSR178GUS
CTCF ChIP-seq on mouse forebrain tissue postnatal (0 days) ENCSR677HXC
CTCF ChIP-seq on mouse midbrain tissue postnatal (0 days) ENCSR985ZTV
CTCF ChIP-seq on mouse hindbrain tissue postnatal (0 days) ENCSR150RGT
CTCF ChIP-seq on mouse heart tissue postnatal (0 days) ENCSR491NUM
CTCF ChIP-seq on mouse liver tissue postnatal (0 days) ENCSR041SMK
CTCF ChIP-seq on mouse intestine tissue postnatal (0 days) ENCSR002ZAG
CTCF ChIP-seq on mouse kidney tissue postnatal (0 days) ENCSR143WOK
CTCF ChIP-seq on mouse lung tissue postnatal (0 days) ENCSR418SBY
CTCF ChIP-seq on mouse stomach tissue postnatal (0 days) ENCSR104QEN
H3K9me3 ChIP-seq on WT(J1) and Dnmt TKO ES cells GEO: GSE29413
CTCF ChIP-seq on WT(J1) and Dnmt TKO ES cells GEO: GSE138102
RNA-seq on WT(J1) and Dnmt TKO ES cells GEO: GSE138102
HP1-α, -β, and -γ ChIP-seq on WT ES cells GEO: GSE97945
KAP-1 ChIP-seq on WT ES cells GEO: GSE58323
RNA-seq on WT and Setdb1 KO ES cells GEO: GSE29413
Bisulfite-seq on WT and Setdb1 KO ES cells GEO: GSE47894
5hmC Capture-seq on WT and Setdb1 KO ES cells GEO: GSE47892
CTCF ChIP-seq on WT and Setdb1 KO neurons GEO: GSE99363
H3K9me3 ChIP-seq on WT and Setdb1 KO neurons GEO: GSE99363
RNA-seq on WT and Setdb1 KO neurons GEO: GSE99363
H3K9me3 ChIP-seq on R1 shLuc ES cells GEO: GSE94086
H3K36me3 ChIP-seq on WT and Setdb1 KO ES cells GEO: GSE171749
CTCF ChIP-seq on Hepatocytes GEO: GSE93431
CTCF ChIP-seq on Th2 cells GEO: GSE66343
SMC3 ChIP-seq on Hepatocytes GEO: GSE93431
SMC1A ChIP-seq on E14 shCtrl and shSetdb1 ES cells GEO: GSE123245
SMC1A ChIP-seq on Th2 cells GEO: GSE66343
RAD21A ChIP-seq on Hepatocytes GEO: GSE93431
SETDB1 ChIP-seq on E14 shCtrl ES cells GEO: GSE123245
SETDB1 ChIP-seq on E14 ES cells GEO: GSE17642
H3K9me3 ChIP-seq on WT and Setdb1 KO ES cells GEO: GSE47894
H3K9me3 ChIP-seq on Th2 cells GEO: GSE66343

Research involving human participants, their data, or biological material

Policy information about studies with human participants or human data. See also policy information about sex, gender (identity/presentation), and sexual orientation and race, ethnicity and racism.

Reporting on sex and gender	Not applicable
Reporting on race, ethnicity, or	Not applicable

other socially relevant groupings	
Population characteristics	Not applicable
Recruitment	Not applicable
Ethics oversight	Not applicable

Note that full information on the approval of the study protocol must also be provided in the manuscript.

Field-specific reporting

Please select the one below that is the best fit for your research. If you are not sure, read the appropriate sections before making your selection.

☒ Life sciences ☐ Behavioural & social sciences ☐ Ecological, evolutionary & environmental sciences

For a reference copy of the document with all sections, see nature.com/documents/nr-reporting-summary-flat.pdf

Life sciences study design

All studies must disclose on these points even when the disclosure is negative.

Sample size	No statistical method was employed to determine sample size. Experiments were conducted on individual cell lines; therefore one biological sample was analyzed per condition.
Data exclusions	No data was excluded from analyses
Replication	For Hi-C experiment, two experimental replicates were conducted independently. Replicates were compared to ensure concordance of datasets. For H3K9me3, CTCF, SMC3 ChIp-seq data, two experimental replicates were conducted independently. All attempts at replication were successful.
Randomization	Randomization is not relevant to this study. No randomization was conducted. Experiments were conducted in mouse embryonic cell line.
Blinding	No blinding was conducted. No human subjects were involved in the study and no subjective measurements were taken.

Reporting for specific materials, systems and methods

We require information from authors about some types of materials, experimental systems and methods used in many studies. Here, indicate whether each material, system or method listed is relevant to your study. If you are not sure if a list item applies to your research, read the appropriate section before selecting a response.

Materials & experimental systems

n/a	Involved in the study	
☐	☒	Antibodies
☐	☒	Eukaryotic cell lines
☒	☐	Palaeontology and archaeology
☒	☐	Animals and other organisms
☒	☐	Clinical data
☒	☐	Dual use research of concern
☒	☐	Plants

Methods

n/a	Involved in the study	
☐	☒	ChIP-seq
☒	☐	Flow cytometry
☒	☐	MRI-based neuroimaging

Antibodies

Antibodies used	ChIP-seq:<br>H3K9me3 (Abcam, 8898) - https://www.abcam.com/products/primary-antibodies/histone-h3-tri-methyl-k9-antibody-chip-grade-ab8898.html<br>H3K27ac (Active motif, 39133) - https://www.activemotif.com/catalog/details/39133/histone-h3-acetyl-lys27-antibody-pab<br>CTCF (Active Motif, 61311) - https://www.activemotif.com/catalog/details/61311/ctcf-antibody-pab<br>SMC3 (Abcam, 9263) - https://www.abcam.com/products/primary-antibodies/smc3-antibody-ab9263.html<br><br>Western blot:<br>SETDB1 (ThermoFisher, PA5-30334) (Dilution 1:1000) - https://www.thermofisher.com/antibody/product/SETDB1-Antibody-Polyclonal/PA5-30334<br>CTCF (Active Motif, 61311) (Dilution 1:1000) - https://www.activemotif.com/catalog/details/61311/ctcf-antibody-pab<br>FLAG (Sigma-Aldrich, F3165) (Dilution 1:1000) - https://www.sigmaaldrich.com/HK/en/product/sigma/f3165#product-documentation

	Tubulin (Abcam, 6046) (Dilution 1:20000) - https://www.abcam.com/products/primary-antibodies/beta-tubulin-antibody-loading-control-ab6046.html
Validation	All antibodies used in this study came with provided certificate of analysis and had prior citations to show their specificity. Peptide arrays have also been carried out to validate specificity of histone modification antibodies.

Eukaryotic cell lines

Policy information about cell lines and Sex and Gender in Research

Cell line source(s)	TT2 mESCs (clone 33#6, C1243A clone#21, and C1243A clone #31) lines. Cell lines were received from the Dr Matthew Lorincz's lab at University of British Columbia.
Authentication	No additional authentication was done
Mycoplasma contamination	Cell lines tested negative for mycoplasma
Commonly misidentified lines (See ICLAC register)	No commonly misidentified cell lines were used in this study.

Plants

Seed stocks	Not applicable
Novel plant genotypes	Not applicable
Authentication	Not applicable

ChIP-seq

Data deposition

☒ Confirm that both raw and final processed data have been deposited in a public database such as GEO.

☒ Confirm that you have deposited or provided access to graph files (e.g. BED files) for the called peaks.

Data access links *May remain private before publication.*	https://www.ncbi.nlm.nih.gov/geo/query/acc.cgi?&acc=GSE184471
Files in database submission	Raw files:<br>ChIPseq_H3K9me3_WT.fastq.gz<br>ChIPseq_H3K9me3_Setdb1KO.fastq.gz<br>ChIPseq_input_H3K9me3_WT.fastq.gz<br>ChIPseq_input_H3K9me3_Setdb1KO.fastq.gz<br>ChIPseq_H3K27ac_WT.fastq.gz<br>ChIPseq_H3K27ac_Setdb1KO.fastq.gz<br>ChIPseq_input_H3K27ac_WT.fastq.gz<br>ChIPseq_input_H3K27ac_Setdb1KO.fastq.gz<br>ChIPseq_CTCF_WT.fastq.gz<br>ChIPseq_CTCF_Setdb1KO.fastq.gz<br>ChIPseq_input_CTCF_WT.fastq.gz<br>ChIPseq_input_CTCF_Setdb1KO.fastq.gz<br>ChIPseq_SMC3_WT.fastq.gz<br>ChIPseq_SMC3_Setdb1KO.fastq.gz<br>ChIPseq_input_SMC3_WT.fastq.gz<br>ChIPseq_input_SMC3_Setdb1KO.fastq.gz<br>ChIPseq_H3K9me3_WT_rep2.fastq.gz<br>ChIPseq_H3K9me3_Setdb1KO_rep2.fastq.gz<br>ChIPseq_input_H3K9me3_WT_rep2.fastq.gz<br>ChIPseq_input_H3K9me3_Setdb1KO_rep2.fastq.gz<br>ChIPseq_CTCF_WT_rep2.fastq.gz<br>ChIPseq_CTCF_Setdb1KO_rep2.fastq.gz<br>ChIPseq_input_CTCF_WT_rep2.fastq.gz<br>ChIPseq_input_CTCF_Setdb1KO_rep2.fastq.gz<br>ChIPseq_SMC3_WT_rep2.fastq.gz

ChIPseq_SMC3_Setdb1KO_rep2.fastq.gz
ChIPseq_input_SMC3_WT_rep2.fastq.gz
ChIPseq_input_SMC3_Setdb1KO_rep2.fastq.gz
ChIPseq_H3K9me3_C21KO.fastq.gz
ChIPseq_H3K9me3_C31KO.fastq.gz
ChIPseq_input_H3K9me3_C21KO.fastq.gz
ChIPseq_input_H3K9me3_C31KO.fastq.gz
ChIPseq_CTCF_C21KO.fastq.gz
ChIPseq_CTCF_C31KO.fastq.gz
ChIPseq_input_CTCF_C21KO.fastq.gz
ChIPseq_input_CTCF_C31KO.fastq.gz
ATACseq_WT.R1.fastq.gz
ATACseq_WT.R2.fastq.gz
ATACseq_Setdb1KO.R1.fastq.gz
ATACseq_Setdb1KO.R2.fastq.gz
MNaseseq_WT.R1.fastq.gz
MNaseseq_WT.R2.fastq.gz
MNaseseq_Setdb1KO.R1.fastq.gz
MNaseseq_Setdb1KO.R2.fastq.gz
HiC_WT_rep1.R1.fastq.gz
HiC_WT_rep1.R2.fastq.gz
HiC_Setdb1KO_rep1.R1.fastq.gz
HiC_Setdb1KO_rep1.R2.fastq.gz
HiC_WT_rep2.R1.fastq.gz
HiC_WT_rep2.R2.fastq.gz
HiC_Setdb1KO_rep2.R1.fastq.gz
HiC_Setdb1KO_rep2.R2.fastq.gz

Processed files :
ChIPseq_H3K9me3_WT_inputsubrpkm.bw
ChIPseq_H3K9me3_Setdb1KO_inputsubrpkm.bw
ChIPseq_H3K27ac_WT_inputsubrpkm.bw
ChIPseq_H3K27ac_Setdb1KO_inputsubrpkm.bw
ChIPseq_CTCF_WT_inputsubrpkm.bw
ChIPseq_CTCF_Setdb1KO_inputsubrpkm.bw
ChIPseq_SMC3_WT_inputsubrpkm.bw
ChIPseq_SMC3_Setdb1KO_inputsubrpkm.bw
ChIPseq_H3K9me3_WT_rep2_inputsubrpkm.bw
ChIPseq_H3K9me3_Setdb1KO_rep2_inputsubrpkm.bw
ChIPseq_CTCF_WT_rep2_inputsubrpkm.bw
ChIPseq_CTCF_Setdb1KO_rep2_inputsubrpkm.bw
ChIPseq_SMC3_WT_rep2_inputsubrpkm.bw
ChIPseq_SMC3_Setdb1KO_rep2_inputsubrpkm.bw
ChIPseq_H3K9me3_C21KO_inputsubrpkm.bw
ChIPseq_H3K9me3_C31KO_inputsubrpkm.bw
ChIPseq_CTCF_C21KO_inputsubrpkm.bw
ChIPseq_CTCF_C31KO_inputsubrpkm.bw
ATACseq_WT_s3normreadscount.bw
ATACseq_Setdb1KO_s3normreadscount.bw
MNaseseq_WT_141to190_wiq_smooth.bw
MNaseseq_Setdb1KO_141to190_wiq_smooth.bw

Genome browser session
(e.g. UCSC)

http://epigenomegateway.wustl.edu/browser/
Session bundle Id: 75140330-66bd-11eb-830a-9fd8fd419734

Methodology

Replicates

ChIP-seq was performed on two biological replicates.

Sequencing depth

ChIP-seq libraries were sequenced on the Illumina NextSeq platform with 75bp reads. All libraries were sequenced to greater than 20 million uniquely mapped reads.

Antibodies

ChIP-seq: H3K9me3 (Abcam, 8898); H3K27ac (Active motif, 39133); CTCF (Active Motif, 61311); SMC3 (Abcam, 9263).

Peak calling parameters

macs2 callpeak --keep-dup all -g 2.43e9 -q 0.05.

Data quality

Peak calls were used for confirmation of data quality. Comparison with previously published datasets from the same cell lines was conducted.

Software

MACS2 v2.1.0
